# Amniotic Fluid Embolism in a Patient Presenting for Induction of Labor for Cholestasis of Pregnancy

**DOI:** 10.7759/cureus.66097

**Published:** 2024-08-03

**Authors:** Emma W Parenteau, Maya Block, David Higgins

**Affiliations:** 1 Anesthesiology, Creighton University School of Medicine, Phoenix, USA; 2 Obstetrics and Gynecology, Creighton University School of Medicine, Phoenix, USA; 3 Anesthesiology, Mountain West Anesthesia, Lehi, USA

**Keywords:** amniotic fluid embolism diagnosis, anesthesiology, amniotic fluid embolism, obstetric anesthesiology, amniotic fluid embolism management

## Abstract

Amniotic fluid embolism (AFE) is a rare but potentially catastrophic complication during pregnancy. The most common presenting symptoms of AFE are cardiac arrest, hemodynamic instability, and profound hemorrhage. Here, we present a case of a 28-year-old female with a past medical history of anemia, gestational diabetes, hypothyroidism, anxiety, and depression, presenting for induction of labor for cholestasis of pregnancy. The labor course was complicated by sudden cardiac arrest and resuscitation, followed by an emergent bedside peri-cardiac arrest cesarean section. Subsequently, the patient developed coagulopathy, uterine atony, and bleeding from the vagina and cervix. The AFE protocol was initiated, and the care team proceeded with a hysterectomy. Supportive treatment correcting for metabolic acid-base disturbances and coagulopathies was maintained. The patient was stabilized for air transport to a higher level of care.

## Introduction

Although rare, the exact incidence of amniotic fluid embolism (AFE) is difficult to determine as the diagnosis and reporting vary [1]. However, various studies estimate an incidence of 1.9-6.1 per 100,000 births in developed countries [2-4]. Patients with AFE may present with a constellation of symptomatology including acute respiratory failure, sudden cardiovascular collapse, and acute onset coagulopathy [3,5]. The complete pathophysiology of AFE is not fully understood. However, it most commonly presents during labor, delivery, and even up to 48 hours postpartum. The management of AFE is largely supportive; however, early identification and aggressive management of the patient's hemodynamics, coagulopathy, and oxygenation are paramount [6].

This article was previously presented as a poster at the 2023 American Society of Anesthesiologists Annual Meeting in San Francisco, California, on October 15, 2023.

## Case presentation

The patient was a 28-year-old gravida 2 para 1 at 37 weeks and 5 days gestation who presented for induction of labor for cholestasis of pregnancy. Pregnancy history was significant for cholestasis, depression, hypothyroidism, anemia, and gestational diabetes mellitus (GDM), managed with metformin. 

Induction was started at 37 weeks and 5 days. The following day, the patient received an epidural at L2-L3 with a negative test dose utilizing lidocaine 1.5% with epinephrine 1-to-200,000. At 07:40, she was evaluated by an obstetrician (OB), and upon physical examination, her cervix was found to be 4 cm dilated. At this time, artificial rupture of membranes (AROM) was performed, an intrauterine pressure catheter (IUPC) was placed, and induction medications were continued.

At 09:05, the patient was found to be vomiting. Shortly after, at 09:14, the patient was found to be unresponsive - pulseless, fetal heart tones were decelerating, and Code Blue was called. The Emergency Room (ER) provider and anesthesiologist arrived at the bedside, the patient was intubated, and CPR was continued. Obstetrics (OB) providers were en route; however, the patient remained in cardiac arrest. During CPR, a general surgeon performed an emergent bedside peri-cardiac arrest cesarean section and delivered the neonate. The neonate presented with the umbilical cord wrapped twice around the neck and without pulses. The nursing and ER team were able to resuscitate the neonate, who was then placed on a continuous positive airway pressure (CPAP) machine. 

Shortly after delivery, the patient received three doses of 1 mg of epinephrine. The patient’s pulse returned, and an arterial line and a right femoral central line were then placed without complications. Intraoperatively, the patient received 15 mg of midazolam, 50 mg of rocuronium, 2 g of cefazolin, 3.375 g of piperacillin-tazobactam, 1 mg of epinephrine, 8 g of calcium chloride 10%, and 850 mEq sodium bicarbonate 8.4% injection. The OB providers arrived to close the uterus, at which point the patient went into cardiac arrest and CPR was resumed. Soon after, the patient resumed spontaneous cardiac activity but was noted to have uterine atony, with blood draining from the vagina and cervix. The AFE protocol was activated, and maternal-fetal medicine (MFM) was consulted. At this time, the patient’s estimated blood loss was approximately 1500 mL. The patient received 30 mg of ketorolac and 8 mg of ondansetron. She was transfused with multiple units of packed red blood cells (PRBCs) and received oxytocin, misoprostol, and tranexamic acid, yet uterine atony persisted. Efforts to control bleeding continued; a Bakri^®^ balloon was placed, and the uterine incision was closed. The patient was noted to have increased oozing from serial edges and continued uterine atony. An additional 200 mL (total 500 mL) of normal saline (NS) was placed in the Bakri balloon. The patient had received four units of PRBCs and was awaiting fresh frozen plasma (FFP). Atropine was held per recommendation from MFM due to current tachycardia.

Uterine atony persisted, and the care team decided to proceed with a hysterectomy. The estimated blood loss at this time was 3500 mL. The patient went into cardiac arrest intraoperatively but resumed spontaneous cardiac activity after chest compressions. An emergent supracervical hysterectomy was performed, and all uterine vessels were properly ligated with extensive cauterization. However, diffuse nonfocal bleeding continued. The patient’s pelvis was packed with laparotomy sponges, and the subcutaneous layer was left open with moist sterile towels. Due to the inability to control the bleeding, interventional radiology then performed an embolization of the left internal iliac artery.

Intraoperatively, the anesthesiologist maintained supportive treatment, correcting metabolic acid-base disturbances and addressing coagulopathy. At this point, the patient’s estimated blood loss was approximately 5000 mL. She had received 16 units of PRBCs, 8 units of FFP, 9 units of cryoprecipitate, and two packs of platelets. Following surgery, the patient was in critical condition but stabilized for air transport to a nearby ICU for a higher level of care. Unfortunately, the patient passed away while in the ICU at the nearby facility.

## Discussion

This is a case of AFE, hemodynamic instability, and cardiac arrest in a 28-year-old patient presenting for induction of labor for cholestasis of pregnancy. This case warrants presentation because of the rarity and severity of AFE, as well as the utilization of emergent care to stabilize the patient. In the present case, the care team was able to stabilize the patient for air transport and preserve the life of the neonate.

It is important to first discuss the current indications for induction of labor for cholestasis of pregnancy. Two retrospective cohort studies suggested that the optimal timing of delivery for patients with cholestasis of pregnancy was at 36 weeks gestation [7]. However, a more recent meta-analysis found a significant difference in the odds of stillbirth prior to 39 weeks gestation only when the serum bile acid levels were >100 μmol/L [8]. The decision to induce the patient presented in the case was based on her concurrent diagnosis of gestational diabetes and cholestasis of pregnancy.

**Figure 1:**
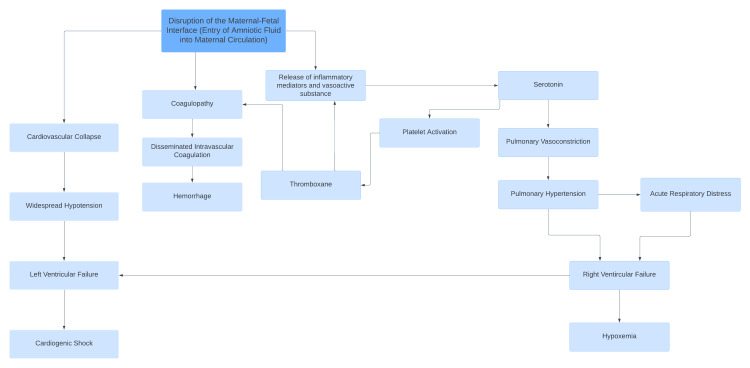
Underlying pathophysiology of amniotic fluid embolism (AFE)

In continuation, the goal of this case study is to demonstrate the importance of early identification and treatment of AFE. Considering the rarity of AFE, a brief discussion of the pathophysiology underlying the case is presented. Although AFE can lead to severe complications, including maternal death and adverse perinatal outcomes, the primary underlying pathophysiology remains a subject of debate. The current understanding is that AFE is triggered by the introduction of fetal cells and debris from the amniotic fluid into the maternal circulation. The introduction of these substances triggers an anaphylactoid reaction [9]. Once in maternal circulation, the foreign fetal material is thought to cause the release of endogenous inflammatory mediators, affecting the clotting cascade as well as cardiovascular and pulmonary function (Figure 1) [3,10-11].

Moreover, it should be recognized that a uniform diagnostic criterion for AFE does not exist. Consequently, AFE remains a diagnosis of exclusion and one based on clinical presentation and presenting symptomatology [12]. AFE classically presents with cardiovascular collapse, respiratory difficulties, and seizures with a classic triad of sudden hypoxia, hypotension, and coagulopathy [3]. Additional abnormalities, including disseminated intravascular coagulation (DIC), hypoxemia, and acidosis, have also been noted [5]. Therefore, utilizing the patient’s clinical presentation of recurrent cardiovascular collapse, uncontrollable hemorrhaging, and hemodynamic instability, as well as the triad of symptoms, the diagnosis of AFE was reached [3,9]. Differential diagnoses that should be considered when diagnosing AFE include anaphylaxis, pulmonary embolism, air embolism, myocardial infarction, septic shock, hemorrhagic shock, eclamptic convulsions, and coma [13].

Due to the severity of AFE, rapid identification and supportive treatment are crucial. AFE frequently presents with sudden cardiac arrest, thus immediate care should include high-quality CPR and a peri-cardiac arrest cesarean section, both of which were performed in our patient [14,15]. The hospital presented in the case is a level-two hospital, thus no in-house OB providers were present at the time of the patient’s cardiac arrest and suspected AFE. Consequently, a cesarean section was performed bedside by a general surgeon with an assisting ER provider.

**Figure 2:**
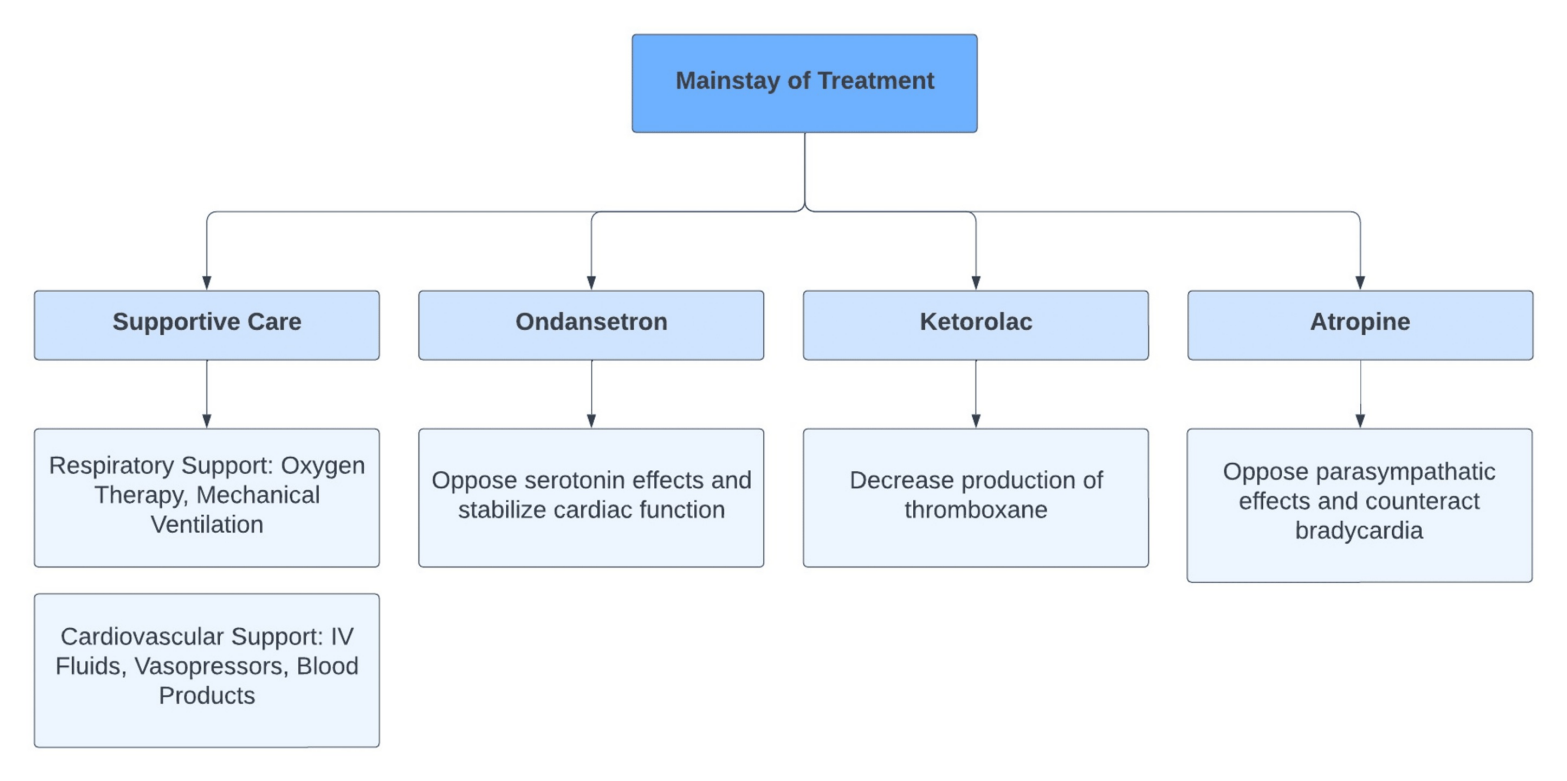
Mainstay of amniotic fluid embolism (AFE) treatment

As previously mentioned, treatment should consist of maintenance of cardiac output and blood pressure, as well as correction of current coagulopathy [6]. In our case, the AFE protocol with ondansetron and ketorolac was initiated. Implementing this protocol alongside cardiac life support has shown positive outcomes in patients with AFE. This is likely due to the proposed mechanisms by which each of these drugs disrupt the underlying pathophysiology of AFE (Figure 2) [15,16]. Additional supportive treatment received by our patient included tranexamic acid, Pitocin, and misoprostol to address uterine atony, as well as PRBCs, FFP, Cryoprecipitate, and platelets to address present coagulopathy.

## Conclusions

This rare case of amniotic fluid embolism (AFE) highlights the importance of early recognition, prompt resuscitation, delivery of the fetus, and maintenance of supportive treatment to improve patient outcomes. The patient’s rapidly worsening clinical status, as indicated by cardiac arrest and decelerating fetal heart tones, demonstrates the critical nature underlying AFE. The lack of a standardized diagnostic criterion for AFE poses significant challenges in quickly identifying and treating AFE. Ultimately, treatment focused on maintaining cardiac output and blood pressure, along with the use of the AFE protocol, which includes ondansetron, ketorolac, and corticosteroids, facilitated the stabilization of the patient for air transport to a higher level of care and preserved the life of the neonate.
